# Asiatic Acid Prevents Retinal Ganglion Cell Apoptosis in a Rat Model of Glaucoma

**DOI:** 10.3389/fnins.2018.00489

**Published:** 2018-07-20

**Authors:** Wanjing Huang, Fengjuan Gao, Fangyuan Hu, Jiancheng Huang, Min Wang, Ping Xu, Rong Zhang, Junyi Chen, Xinghuai Sun, Shenghai Zhang, Jihong Wu

**Affiliations:** ^1^Eye Institute, Eye and ENT Hospital, College of Medicine, Fudan University, Shanghai, China; ^2^State Key Laboratory of Medical Neurobiology, Institutes of Brain Science and Collaborative Innovation Center for Brain Science, Shanghai Medical College, Fudan University, Shanghai, China; ^3^Shanghai Key Laboratory of Visual Impairment and Restoration, Science and Technology Commission of Shanghai Municipality, Shanghai, China; ^4^Key Laboratory of Myopia, Ministry of Health, Shanghai, China; ^5^Department of Ophthalmology, The First Affiliated Hospital of Nanjing Medical University, State Key Laboratory of Reproductive Medicine, Nanjing, China

**Keywords:** glaucoma, retinal ganglion cells, asiatic acid, apoptosis, photopic negative response (PhNR)

## Abstract

Asiatic acid (AA), a pentacyclic triterpene derived from the tropical medicinal plant *Centella asiatica*, has been widely used as an antioxidant and anti-inflammatory agent. Evidence regarding the neuroprotective properties of AA is emerging. However, the protective effects of AA and its mechanism in glaucoma are poorly understood. In the current study, we investigate the neuroprotective effect and mechanism of AA on retinal ganglion cells (RGCs) in a rat model of glaucoma. Elevated intraocular pressure (IOP) was induced in adult rats by injecting microspheres into the anterior chamber. AA was intravitreally injected into glaucomatous rats. RGC densities were analyzed by evaluating surviving RGC number of the retinal flatmounts and retinal sections, and the apoptotic cell number were evaluated by analyzing retinal sections. RGC function was assessed by measuring the photopic negative response (PhNR). Retinal Bcl-2, Bax, and cleaved caspase-3 expression were determined using a Simple Western System, real-time PCR and immunofluorescence staining. AA reduced the loss of RGCs and decreased the apoptotic RGC number. AA exerted neuroprotective effects and ameliorated retinal dysfunction in impaired RGCs in a rat model of glaucoma. AA protected RGCs by upregulating the expression of the antiapoptotic protein Bcl-2 and downregulating the expression of the pro-apoptotic proteins Bax and caspase-3. This study has provided important evidence indicating that AA may be a potential therapeutic agent for glaucoma.

## Introduction

Glaucoma is a leading cause of irreversible vision loss and is characterized by the progressive loss of retinal ganglion cells (RGCs) and their axons (Jonas et al., 2017). The number of patients with glaucoma is estimated to increase to 76 million in 2020 and to 112 million in 2040 (Tham et al., 2014). The major goals of glaucoma research and the clinical treatment of glaucoma are to prevent of progressive RGC degeneration and preserve of the existing visual function.

A variety of complex molecular signals, including mitochondrial dysfunction, glutamate-induced excitotoxic damage, and oxidative stress, are involved in RGC death (Almasieh et al., 2012). Blockade of harmful factors that promote progressive RGC death is an important neuroprotective strategy for glaucoma.

**FIGURE 1:**
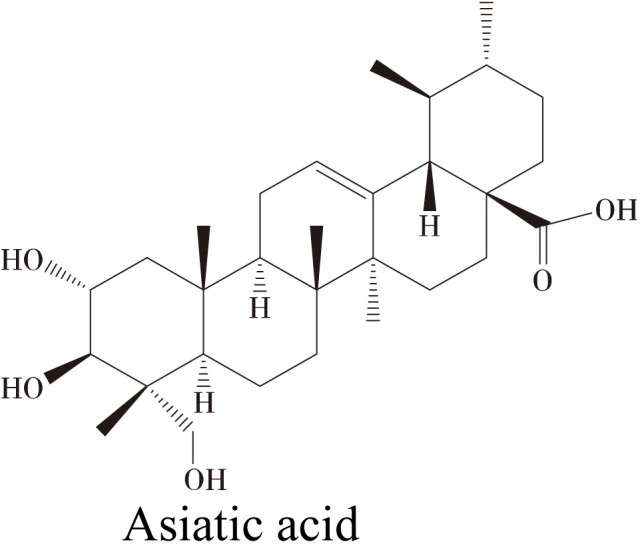
Chemical structure of Asiatic acid (AA).

Asiatic acid (AA) (**Figure 1**) is a pentacyclic triterpene derived from *Centella asiatica* (Umbelliferae) that has been widely used as an antioxidant and anti-inflammatory agent (Lee et al., 2000; Hashim et al., 2011). AA has been reported to protect primary neurons against C_2_-ceramide-induced apoptosis *in vitro* by decreasing cellular reactive oxygen species (ROS) production and maintaining the mitochondrial membrane potential (MMP) (Zhang et al., 2012). AA protects SH-SY5SY cells from rotenone- and H_2_O_2_-induced injury (Xiong et al., 2009) and prevents β-amyloid-induced cell death (Mook-Jung et al., 1999). AA also decreases intracellular free radical concentrations to prevent H_2_O_2_-induced cell death (Jew et al., 2000) and rescues primary rat cortical cells from glutamate-induced toxicity (Lee et al., 2000). AA has been reported to exert neuroprotective effects on focal cerebral ischemia and embolic stroke *in vivo* by preserving the blood–brain barrier (BBB) integrity, reducing the infarct sizes and attenuating mitochondrial damage (Krishnamurthy et al., 2009; Lee et al., 2012, 2014; Tabassum et al., 2013). AA has also been shown to exert robust neuroprotective effects against some neurodegenerative diseases, including Parkinson’s disease and Alzheimer’s disease, both *in vitro* and *in vivo* (Mook-Jung et al., 1999; Jew et al., 2000; Krishnamurthy et al., 2009). However, whether AA also exerts neuroprotective effects on glaucomatous RGC damage is unknown. Oxidative damage appears to be a common component of glaucomatous neurodegeneration (Chen and Kadlubar, 2003; Tezel, 2006). Because AA shows numerous mechanisms that may exert beneficial effects on neurodegenerative diseases, we hypothesized that AA may promote RGC survival in a rat model of glaucoma. In this study, we investigated the neuroprotective effects of AA on RGC apoptosis and function and further explored the underlying mechanisms.

## Materials and Methods

### Animals

All experimental and animal care procedures were performed in accordance with the ARVO Statement for the Use of Animals in Ophthalmic and Vision Research and the guidelines on the ethical use of animals of Fudan University. Experiments were conducted with adult Wistar rats weighing approximately 200–250 g (SLAC Laboratory Animal Co., Ltd., Shanghai, China). Rats were maintained in standard cages under a 12-h light/dark cycle throughout the observation period. A total of 102 Wistar rats were randomly allocated into a normal control (NC) group (*n* = 24), a chronic ocular hypertension (COHT) group (*n* = 24), a COHT+vehicle group (*n* = 24, intravitreous injection of 2 μL of PBS), a COHT+AA group (*n* = 30, intravitreous injection of 2 μL of AA, 1 μmol/L AA, 10 μmol/L AA, 100 μmol/L AA). Rats were euthanized humanely at the indicated time points with an anesthesia overdose by intraperitoneal injection of chloral hydrate (600 mg/kg).

### Ocular Hypertension Model and AA Treatment

Experimental glaucoma was induced by elevating the intraocular pressure (IOP), facilitated by the unilateral injection of 8 μL of paramagnetic microspheres (15 μm, Bangs Laboratories, Fishers, IN, United States) into the anterior chamber, according to the method described by Samsel et al. (2011). A magnet was used to draw the paramagnetic beads into the iridocorneal angle to impede aqueous drainage through the trabecular meshwork. The IOP was measured in anesthetized rats using a TonoLab rebound tonometer (TonoLab, Icare, Vantaa, Finland). Each IOP value represents the mean of five independent measurements. All measurements were performed between 9 and 11 a.m. by the same operator, and each IOP value is reported as the mean ± SD.

Two microliters of AA (Sigma-Aldrich, St. Louis, MO, United States) were intravitreally injected into the appropriate rats when the ocular hypertension model was induced, and then injections were repeated weekly.

### Retrograde Labeling and Quantification of RGCs

After being deeply anesthetized, rats were placed in a stereotactic apparatus. The procedures were performed using 2% hydroxystilbamidine (Fluorogold) (FG, Sigma-Aldrich) in 0.9% NaCl, according to the method described in our previous report (Wu et al., 2015). Briefly, 2 μL of FG were injected into the bilateral superior colliculi at the following coordinates: 6.0 mm posterior to the bregma, 1.2 mm lateral to the midline, and 4.0 mm from the top of the skull. FG was subsequently taken up by the RGC axons and retrogradely transported to the retinal soma. Animals were euthanized, and whole retinas were carefully dissected and flat-mounted at 1 week postinjection. Images were obtained at 1.2–2.0 mm from the optic disk in four quadrants (superior and inferior, nasal and temporal). Four non-overlapping images were captured from the median line of each quadrant, and the number of cells in 16 microscope fields per retina was counted (Wang et al., 2015). RGCs were counted manually using ImageJ software (NIH, Bethesda, MD, United States). Corrections were made for the uptake of FG by microglial cells (Thanos, 1991). Cells displaying a small, irregular rod-shaped or ameboid morphology, which is indicative of microglial cells, were excluded from the total cell counts (Baptiste et al., 2005; Slusar et al., 2013; Yin et al., 2016).

### Histological Evaluation

The methods used for the histological evaluation have been described previously (Sakamoto et al., 2010). Briefly, the eyes were enucleated and fixed with Davidson’s solution (37.5% ethanol, 9.3% paraformaldehyde, and 12.5% acetic acid) for 24 h at room temperature. Fixed eyes were embedded in paraffin after the lenses were removed. Specimens were cut into 5-μm-thick retinal cross-sections and stained with hematoxylin and eosin (H&E) (Sigma-Aldrich). Sections were photographed using a light microscope (Leica, Wetzlar, Germany) and then measured at points located approximately two to three disk diameters from the optic nerve. The number of neurons in retinal ganglion cell layer (GCL) was counted was measured at a point located 1.0–1.5 mm from the optic disk using the method described by Bai et al. (2013). In order to obtain the representative data, we captured and analyzed nine sections per retina and the values of nine sections were averaged to obtain the values for one retina.

### Terminal-Deoxy-Transferase-Mediated dUTP Nick End-Labeling (TUNEL) Assay

TUNEL staining was performed according to the manufacturer’s instructions (*In Situ* Cell Death Detection Kit; Roche, Mannheim, Germany), as described by Wu et al. (2015). Cryosections (10 μm thick) were fixed with 4% paraformaldehyde for 20 min at room temperature and then permeabilized with 0.1% Triton X-100 for 2 min on ice. Samples were subsequently incubated with the TUNEL reaction mixture in a humidified chamber for 60 min at 37°C. After counterstaining with 4’,6’-diamidino-2-phenylindole (DAPI) (1:2000; Life Technologies, Carlsbad, CA, United States), cryosections were visualized under a confocal microscope (Leica SP8, Hamburg, Germany) at 400× magnification. The TUNEL- and DAPI-stained cells were counted as apoptotic cells, and the percentage of TUNEL positive cells were quantified using ImageJ software. Six random fields in each slide were imaged to determine the average percentage of TUNEL positive cells.

### Measurement of the Photopic Negative Response (PhNR)

#### Animal Preparation

The PhNR was measured to evaluate RGC function at 2 weeks after the induction of elevated IOP, as previously reported (Chrysostomou and Crowston, 2013). The PhNR was recorded by an Espion Diagnosys System (Diagnosys, Littleton, MA, United States). After overnight dark adaptation (12 h), PhNR signals were recorded with two 3-mm platinum wire loop electrodes placed on the corneal surface of eyes after the pupils were dilated with phenylephrine hydrochloride and tropicamide (0.5%). A subdermal needle electrode inserted into the base of the right leg served as the ground electrode, and another subdermal needle electrode placed over the nasal bone served as the common reference, as previously described (Zhou et al., 2017). Retinal responses were recorded from both eyes over a duration of 30 min.

#### Recording Protocols

Light stimulation was performed at four stimulus strengths (11.38 candela seconds per meter squared (cd.s/m^2^)–0.33 Hz, 11.38 cd.s/m^2^–1 Hz, 22.76 cd.s/m^2^–0.33 Hz, and 22.76 cd.s/m^2^–1 Hz), using a ColorDome unit from white light-emitted diodes in a four-step test. In each step, the stimulus frequency was 2 Hz, and a 10-cd.s/m^2^ green light was presented against a green background for 4 ms.

#### Waveform Analysis

Waveforms of the PhNR were measured by identifying the maximum peak and trough of the waveforms and measuring the baseline trough and peak amplitude. The values of PhNR amplitudes were compared among the four groups.

### Protein Expression Analysis

A capillary-based “WES” Simple Western System (ProteinSimple, San Jose, CA, United States) was used to quantify the levels of the Bcl-2, Bax, and cleaved caspase-3 proteins, according to the manufacturer’s protocol. Briefly, retinal proteins were extracted using lysis buffer (Cell Signaling Technology, Danvers, MA, United States) supplemented with a protease inhibitor cocktail (Millipore, Billerica, MA, United States), and protein concentrations were determined using a BCA Protein Assay Kit (Thermo Fisher Scientific, Rockford, IL, United States). The Simple Western System allows all post-sample preparation procedures, including the sample loading, size-based protein separation, immunoprobing, washing, detection, and data analysis procedures, to be completed in an automated manner. The following primary antibodies were used for the experiment: mouse anti-β-actin (1:1000 dilution, Abcam, Cambridge, United Kingdom), rabbit anti-Bcl-2 (1:1000 dilution, Abcam), rabbit anti-cleaved caspase-3 (1:1000 dilution, Cell Signaling Technology), and rabbit anti-Bax (1:1000 dilution, Abcam). After the microplates had been loaded, separation and immunoprobing were performed automatically, and the chemiluminescent signals were detected and analyzed by Compass software (ProteinSimple).

### Measurement of mRNA Expression by Quantitative Real-Time PCR (qPCR)

Whole retinas were used for the qPCR analysis. Total RNA was extracted using an RNeasy Mini Kit (Qiagen, Valencia, CA, United States) and reverse transcribed into cDNAs using a PrimeScript RT Reagent Kit (Takara, Tokyo, Japan), according to the manufacturer’s instructions. The qPCR was performed on a Vii 7 instrument (Applied Biosystems, Foster City, CA, United States) with SYBR Premix Ex Taq (Takara). The sequences of the primer used in this experiment were as follows: rat β-actin (forward: 5′-CCGCGAGTACAACCTTCTTG-3′ and reverse: 5′-CAGTTGGTGACAATGCCGTG-3′), rat Bcl-2 (forward: 5′-TTGAGTTCGGTGGGGTCATG-3′ and reverse: 5′-TCAAACAGAGGTCGCATGCT-3′), and rat Bax (forward: 5′-ATTCCGCAGTCTGGGTTAGC-3′ and reverse: 5′-AGTAGGCTCATAACCCTGAG-3′). The reaction consisted of one cycle of 30 s at 95°C followed by 40 cycles of 5 s at 95°C, 30 s at 60°C and 60 s at 72°C. The specificity of the detected signals was confirmed with a curve consisting of a single peak. The data were analyzed using the 2^-ΔΔCT^ method, and relative RNA expression levels were normalized to β-actin expression levels. All samples were analyzed in triplicate in each experiment.

### Immunofluorescence Staining

Immunofluorescence staining was performed as described previously (Wu et al., 2010). Briefly, 10-μm retinal cryosections were fixed with 4% paraformaldehyde for 20 min at room temperature and then incubated with 0.1% Triton X-100 and 3% bovine serum albumin (BSA) for 40 min at room temperature. Sections were then incubated with a rabbit anti-caspase-3 antibody (1:300 dilution, Abcam) overnight before being incubated with an Alexa Fluor 488-conjugated goat anti-rabbit IgG secondary antibody (1:500, Invitrogen-Molecular Probes, Carlsbad, CA, United States) for 1 h at room temperature. Sections were then counterstained with Hoechst 33258 (1:1000, Invitrogen-Molecular Probes) and visualized and photographed using a confocal microscope (Leica SP8).

### Statistical Analysis

Data are presented as mean ± SD. One-way ANOVA with Bonferroni’s multiple comparison test were used to compare the differences in means between different groups, and only variables with a normal distribution were tested. The distributions of the amplitudes and inter-event intervals were compared using the Kolmogorov–Smirnov test. *p* < 0.05 value was considered the threshold of statistical significance.

## Results

### Induction of Experimental Glaucoma

**FIGURE 2:**
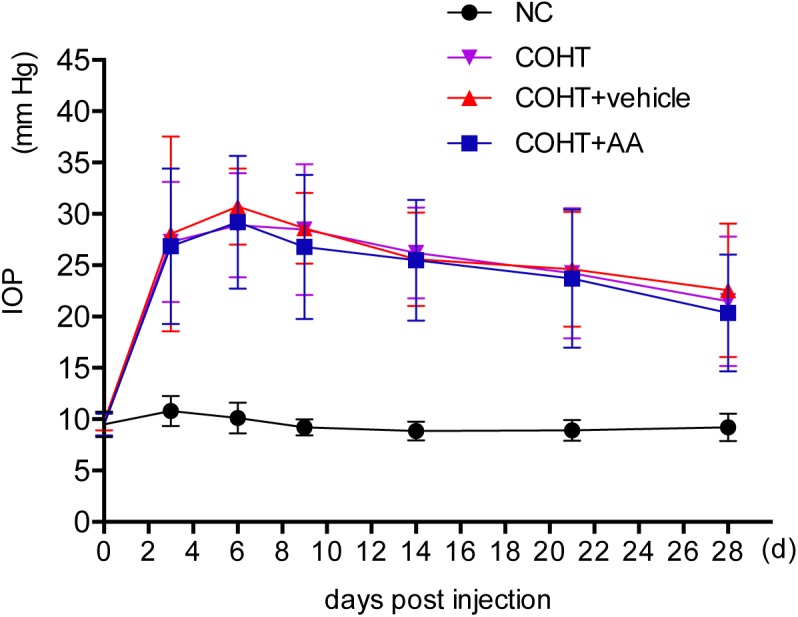
Changes in intraocular pressure (IOP) after the induction of ocular hypertension. The IOP of experimental eyes undergoing paramagnetic microsphere injections were significantly greater than the contralateral control eyes (*n* = 24, *p* < 0.001). The administration of AA did not significantly affect IOP (*n* = 24, *p* > 0.05). IOP values are presented as mean ± SD for each time point.

Chronic ocular hypertension was successfully induced in Wistar rats by the injection of paramagnetic microspheres into the anterior chamber. The IOP was significantly elevated (defined as 5-mmHg higher than the corresponding pressure in the contralateral eye) (Chan et al., 2007) on the third day after the microbead injection (27.27 ± 5.86 mmHg in the COHT group compared with 10.80 ± 1.47 mmHg in the NC group, *n* = 24, *p* < 0.001). The IOP in the microbead-injected eye remained significantly higher than the IOP in the NC group at each time point analyzed over the 4-week period (**Figure 2**). In addition, AA administration did not affect the IOP (26.86 ± 7.57 mmHg in the COHT+AA group compared with 27.27 ± 5.86 mmHg in the COHT group, *n* = 24, *p* = 0.852, >0.05).

### AA Increases RGC Survival in the Experimental Rat Model of Glaucoma

**FIGURE 3:**
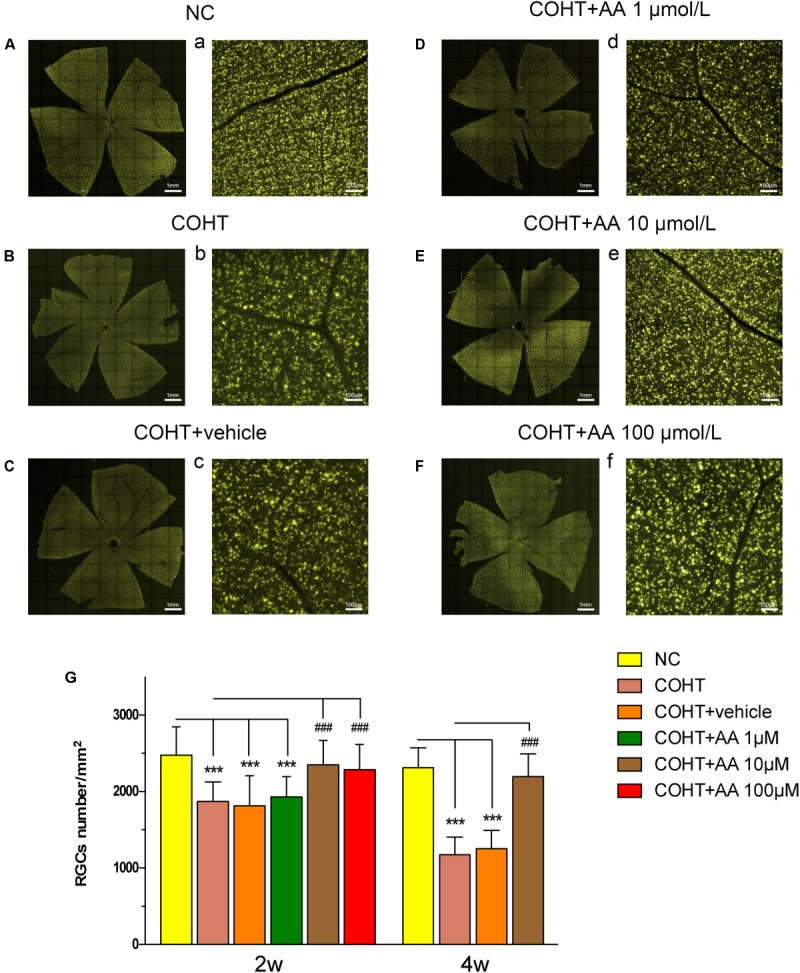
Protective effect of AA on FG-labeled RGCs after the induction of elevated IOP. **(A–F,a–f)** Representative images of FG-labeled surviving RGCs in the retinal flatmounts from the NC, COHT, COHT+vehicle and AA groups captured 2 weeks after the establishment of ocular hypertension. Images were captured at the same magnification. **(G)** Quantitative analysis of FG-labeled RGC densities in the NC, COHT, COHT+vehicle, and COHT+AA (1, 10, and 100 μM) groups (*n* = 10) after 2 and 4 weeks. Data are presented as mean ± SD, ^###^*p* < 0.001, compared to the COHT group. ^∗∗∗^*p* < 0.001, compared to the NC group.

We counted the number of FG-labeled RGCs in retinal flatmounts to assess whether AA increased RGC survival in a model of COHT. We identified the bright and diffuse gold fluorescence of the FG-labeled RGCs in the NC and COHT+AA groups. However, the gold fluorescence was typically weaker in the COHT and COHT+vehicle groups than in the aforementioned groups (**Figures 3A—F,a–f**). Consistent with the results of our previous studies (Wu et al., 2010), the number of RGCs was significantly decreased in the COHT group compared to the NC group (**Figure 3**). The mean RGC densities in the COHT and COHT+vehicle groups were 1871.43 ± 252.03 cells/mm^2^ (mean ± SD) and 1813.19 ± 392.94 cells/mm^2^ at 2 weeks and 1169 ± 229.0 cells/mm^2^ and 1242 ± 239.73 cells/mm^2^ at 4 weeks, respectively. Based on these findings, the RGC density was decreased by approximately 24.51 and 26.86% at 2 weeks and 49.39 and 45.91% at 4 weeks in the COHT and COHT+vehicle groups, respectively, after IOP elevation.

However, the mean RGC densities in the COHT+AA groups (10 and 100 μmol/L) were 2350.30 ± 318.54 and 2285 ± 332.83 cells/mm^2^ (*n* = 10, *p* > 0.05, compared to the NC group) at 2 weeks, respectively. Thus, the RGC density decreased by only 5.19 ± 12.85 and 5.85 ± 13.93% in the 10 and 100 μmol/L AA groups compared to the NC group (2479.00 ± 368.92 cells/mm^2^, *n* = 10), respectively (**Figure 3G**). However, low AA concentrations (1 μmol/L) did not significantly increase the number of surviving RGCs, as the cell density in the indicated group was 1928.18 ± 266.91 cells/mm^2^ (*p* > 0.05). Based on these data, AA increases RGC survival after the induction of elevated IOP. We elected to use 10 μmol/L AA in subsequent experiments because the dose exerted similar protective effects to 100 μmol/L AA (*p* > 0.05).

**FIGURE 4:**
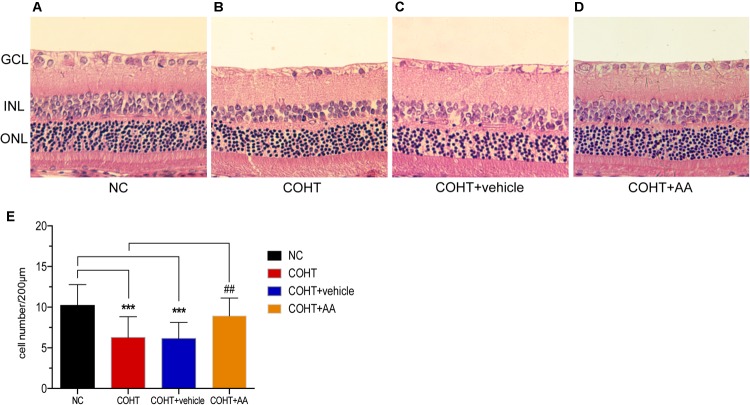
Histological differences observed across NC, COHT, COHT+vehicle, and COHT+AA groups at 2 weeks. **(A–D)** Representative images of H&E-stained retinal cross-sections from the NC, COHT, COHT+vehicle, and COHT+AA groups (*n* = 6 per group). Scale bar: 25 μm. **(E)** Quantification of the number of cells in the GCL. Data are presented as mean ± SD, ^##^*p* < 0.01, ^###^*p* < 0.001, compared to the COHT group; ^∗∗∗^*p* < 0.001, compared to the NC group (*n* = 6 per group). GCL, ganglion cell layer; INL, inner nuclear layer; ONL, outer nuclear layer.

Histological changes were evaluated by performing H&E staining of retinal cross-sections (**Figures 4A–D**). As shown in **Figure 4A**, the GCL of the NC group displayed a normal structure and standard thickness and contained a large number of cells. In contrast, as shown in **Figures 4B,C**, the GCL of the COHT and COHT+vehicle group were thinner and contained fewer cells. The densities of H&E-stained cells in the GCL at 2 weeks were 10.23 ± 2.54 cells/200 μm (*n* = 6, *p* < 0.001, compared to the COHT group), 6.28 ± 2.54 cells/200 μm (*n* = 6, *p* < 0.001, compared to the NC group), 6.13 ± 2.00 cells/200 μm (*n* = 6, *p* < 0.001, compared to the NC group; *p* = 0.87, compared to the COHT group), and 8.92 ± 2.20 cells/200 μm (*n* = 6, *p* = 0.115, compared to the NC group; *p* = 0.0017, compared to the COHT group) in the NC, COHT, COHT+vehicle, and COHT+AA groups, respectively (**Figure 4E**). AA exerted significant protective effects by increasing the number of surviving RGCs in the AA-treated group compared to the vehicle-treated group. These findings demonstrated that AA prevented harmful changes in retinal thickness after ocular hypertension and increased surviving RGC number.

### AA Attenuated RGC Apoptosis in Rats With COHT

**FIGURE 5:**
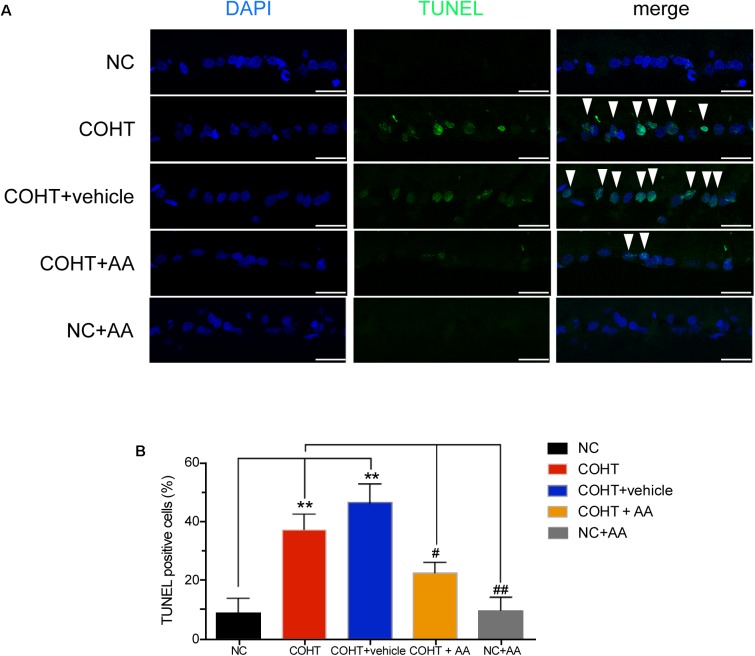
TUNEL staining of the five groups 2 weeks after induction of COHT. **(A)** Images of TUNEL staining in the GCL of retinal sections. The NC group showed a normal GCL without TUNEL-positive cells. The COHT and COHT+vehicle groups showed a large number of TUNEL-positive cells. The COHT+AA and NC+AA groups showed lower number of TUNEL-positive cells in the GCL. Scale bar: 25 μm. **(B)** Quantitative analysis of percentages of TUNEL-positive cells in the GCL at 2 weeks. Data are presented as mean ± SEM. ^#^*p* < 0.05, ^##^*p* < 0.01, compared to the COHT group; ^∗∗^*p* < 0.01, compared to the NC group (*n* = 6 per group).

We assessed RGC apoptosis by performing TUNEL staining of retinal sections 2 weeks after the induction of COHT. RGCs in the NC group were essentially negative for TUNEL staining. In contrast, many TUNEL-positive cells were observed in the GCL of the COHT and COHT+vehicle groups. However, only a few TUNEL-positive cells were observed in the COHT+AA group (**Figure 5A**). The percentage of TUNEL-positive cells in the NC, COHT, COHT+vehicle, and COHT+AA groups were 8.80 ± 4.97% (mean ± SEM) (*n* = 6, *p* = 0.0038, <0.05, compared to the COHT group), 37.00 ± 5.27% (*n* = 6, *p* = 0.0038, <0.05, compared to the NC group), 46.16 ± 6.50% (*n* = 6, *p* = 0.0019, <0.05, compared to the NC group; *p* = 0.3014, compared to the COHT group) and 22.29 ± 3.52% (*n* = 6, *p* = 0.058, compared to the NC group; *p* = 0.028, <0.05, compared to the COHT group) at 2 weeks, respectively (**Figure 5B**). The intravitreal injection of AA (10 μmol/L) significantly reduced RGC apoptosis number after the induction of ocular hypertension. We intravitreally injected AA into the normal rat retina to confirm the safety of AA. The percentage of TUNEL-positive cells in the NC+AA retina was not significantly different from the NC group (9.6 ± 4.434%, *n* = 6, *p* = 0.907, compared to the NC group; *p* = 0.0038, <0.01, compared to the COHT group).

### AA Administration Ameliorates Retinal Dysfunction in a Rat Model of Experimental Glaucoma

**FIGURE 6:**
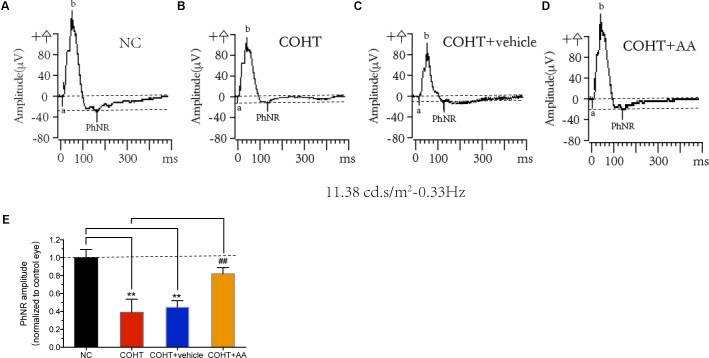
Effects of AA on photopic negative response (PhNR). **(A–D)** Representative PhNR amplitudes in NC, COHT, COHT+vehicle, and COHT+AA groups, respectively. **(E)** Quantitative analysis of PhNR amplitudes in the four groups at 2 weeks (*n* = 12 per group). Amplitudes were normalized to the amplitude of the NC group. Data are presented as mean ± SD, ^##^*p* < 0.01, compared to the COHT group; ^∗∗^*p* < 0.01, compared to the NC group.

Retinal ganglion cell dysfunction precedes RGC death, and RGC dysfunction may be reversible at the early stage of RGC injury (Porciatti, 2015). We analyzed the PhNR, a sensitive marker of inner retinal layer function in patients with glaucoma (Preiser et al., 2013; ElGohary and Elshazly, 2015; Kirkiewicz et al., 2016), to determine whether AA improved retinal function in a rat model of experimental glaucoma (**Figures 6A–D**). PhNR amplitudes were significantly reduced by 63.22 ± 13.89% (mean ± SD) and 57.88 ± 6.95% in the COHT eyes and COHT+vehicle eyes compared to NC eyes at 2 weeks (**Figure 6E**). AA administration attenuated the reductions in PhNR amplitudes in COHT eyes, as PhNR amplitudes in the AA-treated group were only 22.56 ± 6.44% lower than those in the NC group (*n* = 12, *p* = 0.01, <0.05, compared to the COHT group). These results showed that AA can lessen retinal dysfunction in a rat model of experimental glaucoma.

### Mechanisms Underlying the Neuroprotective Effect of AA

**FIGURE 7:**
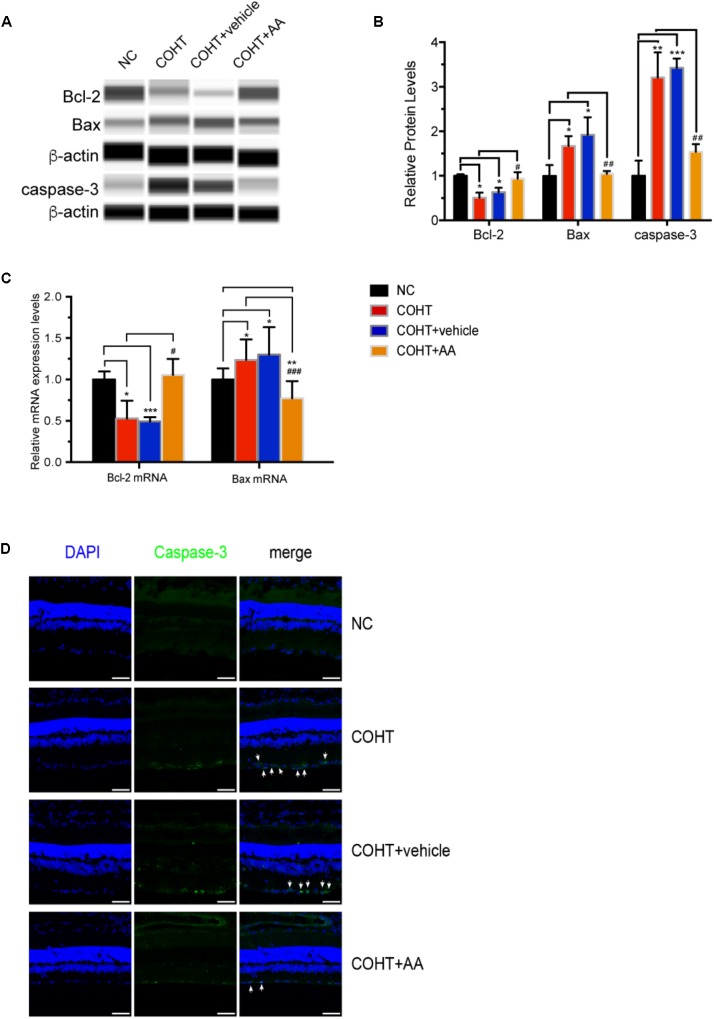
Expression of the antiapoptotic protein Bcl-2 and pro-apoptotic proteins Bax and cleaved caspase-3 among the four groups 2 weeks after establishment of the chronic glaucomatous model. **(A)** Representative digital images of immunoblots of the three apoptosis-related proteins, Bcl-2, Bax, and cleaved caspase-3, were captured using the WES Simple Western System (ProteinSimple). **(B)** Relative protein levels of Bcl-2, Bax, and cleaved caspase-3. Data are presented as mean ± SD. ^#^*p* < 0.05, ^##^*p* < 0.01, compared to the COHT group. ^∗^*p* < 0.05, ^∗∗^*p* < 0.01, ^∗∗∗^*p* < 0.001, compared to the NC group. The protein expression level in the NC group was set to 1. **(C)** Relative mRNA expression levels of Bcl-2 and Bax in NC, COHT, COHT +vehicle, and COHT+AA groups. Data are presented as mean ± SD. ^#^*p* < 0.05, ^###^*p* < 0.001, compared to the COHT group. ^∗^*p* < 0.05, ^∗∗^*p* < 0.01, ^∗∗∗^*p* < 0.001, compared to the NC group. **(D)** Representative images of immunofluorescence staining for caspase-3 (green) and DAPI (blue) in retinal sections from the four groups. Arrows indicate the expression of the pro-apoptotic protein cleaved caspase-3. Scale bar: 50 μm.

We analyzed the expression of antiapoptotic and pro-apoptotic proteins to further investigate the mechanisms underlying the protective effect of AA on ocular hypertension. As shown in **Figures 7A,B**, the expression of the antiapoptotic protein Bcl-2 was decreased (*n* = 6, *p* = 0.002, <0.05, compared to the NC group), and the expression of the pro-apoptotic proteins Bax and cleaved caspase-3 was increased (*n* = 6, *p* = 0.0237, <0.05, compared to the NC group; *n* = 6, *p* = 0.0044, <0.05, compared to the NC group) in the COHT group. However, these changes were reversed by the AA treatment (*n* = 6, *p* = 0.0151, <0.05, compared with the COHT group; *p* = 0.0091, <0.05, compared with the COHT group; *n* = 6, *p* = 0.0081, <0.05, compared with the COHT group).

The expression of the Bcl-2 and Bax mRNAs were also analyzed (**Figure 7C**). As expected, Bcl-2 expression in the COHT and COHT+vehicle groups decreased significantly to levels that were 0.53 ± 0.2- and 0.49 ± 0.05-fold of the levels in the NC group (*n* = 6, *p* = 0.0193, <0.05, compared to the NC group; *n* = 6, *p* < 0.001, compared to the NC group). AA upregulated the expression of the Bcl-2 mRNA in the AA-treated group to a level that was 1.05 ± 0.19-fold of the level in the NC group (*n* = 6, *p* = 0.64, >0.05, compared to the NC group; *p* = 0.034, <0.05, compared to the COHT group). In addition, Bax mRNA expression in the COHT and COHT+vehicle groups increased to levels that were 1.23 ± 0.24- and 1.30 ± 0.33-fold higher than those in the NC group (*n* = 6, *p* = 0.011, <0.05, compared to the NC group; *n* = 6, *p* = 0.015, <0.05, compared to the NC group, respectively), respectively. However, the AA treatment downregulated Bax mRNA expression in the AA-treated group to a level that was 0.77 ± 0.20-fold of the level in the NC group (*n* = 6, *p* = 0.005, <0.05, compared to the NC group; *p* < 0.001, compared to the COHT group).

We analyzed the expression of caspase-3, a pro-apoptotic protein, using immunofluorescence staining to further assess the protective effects of AA on a rat model of experimental glaucoma. Caspase-3 was abundantly expressed in the GCLs of the COHT and COHT+vehicle groups but was expressed at very low levels in the GCL of the NC and COHT+AA groups (**Figure 7D**).

Based on these findings, AA protects RGCs from ocular hypertension by upregulating the expression of the antiapoptotic marker Bcl-2 and downregulating the expression of the pro-apoptotic markers Bax and caspase-3.

## Discussion

The results of this study provided evidence that AA exerted protective effects in a rat model of experimental glaucoma. AA increased RGC survival and function in a rat model of COHT. Moreover, AA effectively prevented RGC apoptosis by upregulating the expression of Bcl-2 and downregulating the expression of Bax and caspase-3.

Glaucoma is the most frequent cause of irreversible blindness and is characterized by progressive RGC loss (Jonas et al., 2017). The current glaucoma therapies aim to reduce IOP; however, neuroprotection may be an effective strategy for treating glaucoma since RGC death is the cause of irreversible vision loss (Yang et al., 2016; Kimura et al., 2017). For example, α-lipoic acid treatment prevents RGC death in glaucomatous retinas in DBA/2J mice, and the administration of another antioxidant, tempol, protects RGCs by limiting neuroinflammation (Yang et al., 2016). In the rat model of glaucoma, treatment with a *Ginkgo biloba* extract (a nitric oxide scavenger) protects RGCs, whereas dietary deficiencies in antioxidants increase RGC loss (Hirooka et al., 2004; Ko et al., 2010). Treatments that reduce oxidative stress in RGCs may be a novel therapeutic strategy for glaucoma; however, no effective neuroprotectants are available to be applied as a treatment for glaucoma. AA was reported to possess antioxidant, anti-inflammatory, and antiexcitotoxic properties and to improve mitochondrial function (Huang et al., 2016; Chen et al., 2017; Nataraj et al., 2017) and is thus believed to target multiple mechanisms underlying glaucoma development. In humans, elevated IOP is the major risk factor for glaucoma. We established a model of glaucoma by injecting magnetic microbeads into anterior chamber to elevate IOP and further investigated the effect of AA (Samsel et al., 2011). In our study, the RGC apoptosis induced by elevated IOP was reversed by the AA treatment.

Retrograde labeling is a reliable method of RGC labeling to identify the population of RGC. FG or its analog hydroxystilbamidine methanesulfonate (OHSt) which is a small molecule with similar fluorescent are the tracers of choice in many laboratories because they are efficient and reliable within the visual system (Sanchez-Migallon et al., 2011; Vidal-Sanz et al., 2012). It is documented that approximately 98.4 and 97.8% of the total RGC population can be labeled in adult albino and pigmented rats (Salinas-Navarro et al., 2009). Some RGC markers (typically a protein expresses only in RGC and continue to express before and after injury) such as γ-synuclein, Brn3a, Thy1 can be used to label RGC (Nuschke et al., 2015). However, retrograde labeling is an unbiased, reliable reproducible way to assess the RGC population from retina to retina in rats and mice, with a level of accuracy, consistency and similarity of the result hardly attained with other methods (Salinas-Navarro et al., 2009).

Consistent with the results of previous studies, RGC numbers were decreased, and the thickness of GCL was reduced in rats with COHT (Russo et al., 2015; Lambuk et al., 2017). In the present study, AA increased the number of surviving RGCs and maintained the normal thickness of the GCL. Additionally, TUNEL staining showed that AA obviously reduced the percentage of apoptotic cells in a rat model of experimental glaucoma. The recovery of RGC function plays the most significant role in neuroprotection. RGC dysfunction occurs early and precedes the loss of optic tissues and the decrease in RGC density (Porciatti, 2015). The PhNR is reported to originate in the inner retinal layer and is detected invasively (Wilsey and Fortune, 2016). The PhNR amplitudes are well correlated with the thickness of the ganglion cell complex within the central macula and provide a direct, objective assessment of the changes in RGC function (Wilsey and Fortune, 2016). In the present study, the PhNR amplitudes in the COHT and COHT+vehicle groups were significantly reduced compared with the NC group; however, the AA treatment ameliorated RGC dysfunction. However, few neuroprotectants have been shown to be successfully rescue RGC function (Biermann et al., 2010; Kyung et al., 2015; Russo et al., 2015). Importantly, AA not only attenuated RGC apoptosis but also successfully ameliorated RGC dysfunction in the present study.

Furthermore, our study revealed that AA effectively prevented RGC apoptosis by upregulating the expression of the antiapoptotic protein Bcl-2 and downregulating the expression of the pro-apoptotic proteins Bax and caspase-3. The mitochondrial pathway leading to caspase activation is the general pathway involved in the cellular response to apoptosis. In addition, the Bcl-2 family, which comprises pro- and antiapoptotic proteins, is a key regulator of the latter process (Granville and Gottlieb, 2002; Wang et al., 2013). The activation and oligomerization of Bax results in the formation of a voltage-dependent anion channel (VDAC)-containing pore or the permeabilization of the mitochondrial membranes to initiate cytochrome c release (Wei et al., 2001), which activates downstream effector caspases. Progressive caspase-independent mitochondrial dysfunction has been reported to induce cell death (Mootha et al., 2001). The activation of caspase-3, downregulation of Bcl-2 and upregulation of Bax in a rat model of glaucoma were reversed by AA, indicating that AA inhibits RGC apoptosis by regulating the mitochondrial pathway-related cell death.

One of the limitations of this study was that it did not completely elucidate the mechanism by which AA protects RGCs. AA has been reported to maintain the MMP, inhibit the elevation of VDAC, decrease the cellular production of ROS and reduce the release of cytochrome c and apoptosis-inducing factors from the mitochondria in some neurodegenerative diseases (Xiong et al., 2009; Lee et al., 2014; Nataraj et al., 2017). Additional in-depth studies are required to further elucidate the precise pathway underlying the therapeutic effects of AA. In addition, an *in vitro* study is required to study the mechanism underlying the effects of AA on primary purified RGCs.

## Conclusion

This study demonstrates the neuroprotective effect of AA in a rat experimental glaucomatous model. AA is a natural small-molecule extract that offers the advantages of easy absorption. AA exerted beneficial effects in neuroprotection by decreasing RGC apoptosis and rescuing retinal dysfunction. AA may be a useful treatment for glaucoma, and additional studies regarding this possibility are ongoing.

## Author Contributions

WH, SZ, and XS designed this work, revised it critically, and finally approved the version to be published. FG, FH, JH, PX, RZ, JC, and MW took part in some of the experimental design and operation, for example, western blotting, PCR analysis, and the rat model establishment. JW drafted, revised the manuscript and took part in a majority of the work. All authors read and approved the manuscript.

## Conflict of Interest Statement

The authors declare that the research was conducted in the absence of any commercial or financial relationships that could be construed as a potential conflict of interest.
